# A Novel Near-Surface Wave-Coherent Instantaneous Profiling System for Atmospheric Measurements

**DOI:** 10.3390/s23084099

**Published:** 2023-04-19

**Authors:** Mathew J. Stanek, Douglas M. Pastore, Erin E. Hackett

**Affiliations:** Department of Marine Science, Coastal Carolina University, Conway, SC 29526, USA

**Keywords:** marine atmospheric boundary layer, air temperature, humidity, ocean surface waves, air–sea interface, in-situ measurements

## Abstract

Large knowledge gaps concerning the effect of ocean surface waves on near-surface vertical distributions of temperature and humidity exist due to practical limitations and sensor fidelity challenges of direct measurements. Measurements of temperature and humidity are classically made using rocket- or radiosondes and fixed weather stations and can utilize a tethered profiling system. However, these measurement systems have limitations when obtaining wave-coherent measurements near the sea surface. Consequently, boundary layer similarity models are commonly employed to fill in near-surface measurement gaps despite the documented shortcomings of the models in this region. Thus, this manuscript presents a near-surface wave-coherent measurement platform that measures high-temporal-resolution vertical distributions of temperature and humidity down to ~0.3 m above the instantaneous sea surface. The design of the platform is described along with preliminary observations obtained during a pilot experiment. Ocean surface-wave phase-resolved vertical profiles are also demonstrated from the observations.

## 1. Introduction

Air–sea interactions and boundary layer turbulence cause the region just above the sea surface in the lowest portion of the marine atmospheric surface layer (MASL) to be extremely complex and present a number of challenges for direct measurements in this region. This region is characterized by large exchanges of momentum, heat, and water vapor with varying spatiotemporal scales. The presence of a non-stationary, moving boundary—i.e., ocean surface waves—further complicates this region, modifying both the transfer of heat and the mechanical mixing through a variety of processes including the effects of sea-spray and skin sea-surface temperature inhomogeneities. Currently, it is not well understood how near-surface heat and water vapor distributions are impacted by ocean surface waves. This lack of understanding is a consequence of the fully coupled nature of the ocean with the atmosphere.

Within the MASL, vertical distributions of temperature and humidity profiles are commonly modeled utilizing Monin Obukhov (MO) similarity theory or similar bulk parametrization algorithms. Two major reported shortcomings of this theory are: (i) the validity of assumptions regarding constant heat and water vapor fluxes [1] and (ii) the neglect of effects due to the ocean-free surface (non-stationary surface [2,3]).

One of the issues that continues to limit further development of empirical and theoretical relationships addressing knowledge gaps in this region is a lack of in-situ near-surface data. Most previous measurements of heat and moisture over marine surfaces are performed using rocket or radiosondes and fixed weather stations, including the use of tethered balloon profiling systems (e.g., marine atmospheric profiling system used during the Coupled Air–sea Processes and Electromagnetic Ducting Research East field campaign [4]). These measurement systems are not able to perform wave-coherent measurements in the near-surface region. For example, these systems can be limited by bulky sensor housings, or utilize profiling techniques that cannot measure instantaneous profiles (O(1s)).

Very limited wave-coherent and/or fine spatial resolution measurements of near-surface humidity and temperature have been made to provide insight into heat and moisture exchange near the air–sea interface, where prior vertical profile measurements are limited in number of levels observed [5]. Many previous studies construct vertical profiles with less than four (vertical) measurements [6,7,8,9,10]. Consequently, there is still a need for the development of a wave-coherent surface measurement platform that is suited to perform measurements across a wide range of wind and wave conditions [11].

This manuscript describes the development and evaluation of a novel near-surface wave-coherent instantaneous profiling system (NWIPS) and also demonstrates preliminary data on the influence of sea surface waves on vertical distributions of temperature and humidity. NWIPS incorporates 30 temperature and humidity sensors, vertically spaced 0.10 m apart, and when combined with a sea-surface temperature probe, comprise a high-resolution scalar profile spanning from the surface to ~3 m in altitude. These measurements are placed in a better context with supplementary measurements made with Windsonds at four altitudes above 3 m but below 15 m. NWIPS measurements are evaluated against measurements made by a weather station, a commonly used benchmark (Vaisala WXT536).

Although only a short time series (80 min) was acquired from NWIPS in the pilot experiment, it is the first attempt to obtain high-resolution, wave-coherent vertical measurements of temperature and humidity. NWIPS is a low-cost platform, which utilizes simple, inexpensive, and off-the-shelf sensors and electronics for the mast. The low cost of onboard sensors means that they can be replaced multiple times during a deployment as inevitably damaged by sea spray and sea salt [12]. In addition, it implements lightweight and cost-effective materials allowing for easy transport and deployment from smaller vessels. Many NWIPS features attempt to address some of the shortcomings of previously implemented marine surface layer measurement platforms, for example, a lack of high vertical spatial resolution [5] and measurements not extending to the sea surface (below ~0.5 m; [4,5,13]). Furthermore, this manuscript illustrates proof of the concept of NWIPS and that measurements of this type are attainable where future modifications of the NWIPS system will improve upon the limitations and shortcomings.

The structure of this paper is as follows: In Section 2, a technical description of the design of NWIPS and the pilot experiment are described. Section 3 demonstrates wave-coherent measurements acquired with NWIPS. Lastly, Section 4 provides a brief summary and discussion.

## 2. Materials and Methods

### 2.1. NWIPS Design

**Figure 1:**
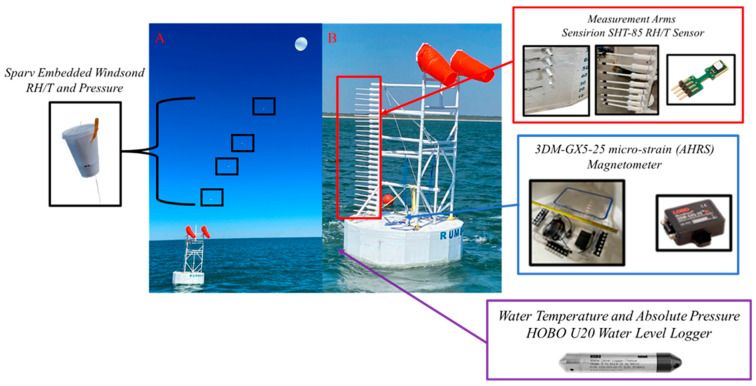
Image of the NWIPS and associated sensors. Panel (**A**) shows NWIPS deployed, and Panel (**B**) is a zoom-in of the buoy and mast from Panel (**A**).

The near-surface wave-coherent instantaneous profiling system (NWIPS) is illustrated in Figure 1. This platform is a custom-designed large discus-type buoy based on the National Oceanic and Atmospheric Administration National Data Buoy Center’s 3 m discus buoy [14]. The buoy implements lightweight and cost-effective materials. The platform incorporates a vertical array of small temperature and humidity sensors spanning 0–3 m above the instantaneous sea surface. With measurements approximately every 10 cm over this interval, the vertical resolution is high compared to other buoys [15] and platform studies, such as those that use R/P FLIP [5]. In addition, NWIPS incorporates a stern-mounted tethered weather balloon with miniature radiosonde systems (Windsonds), providing four additional upper altitude measurements (Figure 1A).

The hull is constructed of ¾ inch plywood, which is kerf cut to provide a hexadecagon (very close to circular) shape. Three layers of 1.5 oz fiberglass are used to fortify the wooden structure and ensure a robust watertight hull. The buoy has a hull diameter of 2.4 m, a depth of 0.75 m, and a draft of ~0.10 m (481 kg) where the overall design provides a 0.65 m freeboard depth. The center of the mass is 0.63 m on-center above the bottom of the buoy and the center of the buoyancy lies at the geometric center of the displaced amount of water which is ~0.05 m above the buoy’s bottom.

The NWIPS’s mast is constructed from polyvinyl chloride (PVC) extending from below the buoy deck (i.e., from within the hull) to ~3 m above the buoy deck. PVC is utilized as the mast material because it is white, as well as being lightweight and rigid. White is chosen as the primary color of the buoy platform and mast to minimize surface heating of the structure caused by shortwave radiation. The mast is constructed in a triangular truss to promote stability where measurement arms protrude from the forward mast and extend outward radially from the buoy. The overall length of a measurement arm is 0.63 m where ~0.12 m extends over the water surface. The lowest measurement position is 0.27 m above the instantaneous water level with a sensor every 0.10 m above this point up to 3.0 m altitude. The lowest four measurement arms protrude out from the buoy hull to obtain measurements as close to the sea surface as possible (Figure 1B—see inset). Each measurement arm houses an SHT-85 sensor, which measures temperature and humidity. This sensor has previously been used as a calibration sensor on unmanned aircraft systems [16]. Side-by-side comparisons of this sensor with a MaxiMet weather station show similar performance under a range of field and lab conditions [17]. Above the mast, a weather balloon is attached with Windsonds nominally located at 7 m, 10 m, 12 m, and 15 m above mean water level (MWL). However, the Windsond altitudes vary (at or below the nominal altitude) as a result of the movement of the balloon caused by the wind (Figure 1A); thus, their exact altitude for each temperature/humidity measurement is measured via pressure by each Windsond. Below the mast and buoy hull, a HOBO U20 water level data logger is used to obtain the bulk sea-surface temperatures and absolute pressure, and is mounted at a depth of ~0.27 m below the instantaneous water level immediately below and forward of the buoy hull.

To ensure thermodynamic variable measurements are not disturbed by the platform wake, two windsocks are positioned atop each of the stern vertical masts self-orienting the buoy and measurement mast into the wind. This positioning minimizes mast structure interference on measurements and further aids the reduction in both thermal and flow distortions (e.g., [18,19]). Buoy orientation is determined via the 3DM-GX5-25 micro-strain attitude heading reference system (AHRS; Lord Parker), mounted within the buoy hull. For the experiment, a Vaisala WXT536 weather station was located aboard the R/V Coastal Explorer (CE), which was the vessel used to deploy the buoy, and its measurements are compared to the buoy heading obtained by the AHRS to verify the proper orientation of the mast and also for verification of the accuracy of meteorological measurements.

NWIPS SHT-85 sensor integration is accomplished through the implementation of a series of watertight electronic boxes housed in the interior of the hull. Each box contains an Arduino Uno microcontroller, a datalogging shield with a real-time clock, and an inter-integrated circuit (I^2^C) multiplexer (TCA9548A by Adafruit). The I^2^C multiplexer allows multiple SHT-85 sensors, which have the same serial address, to operate simultaneously. Prior to deployment, all electronic boxes are initialized, and time stamps are synchronized to a central time with a digital clock onboard the R/V CE. The AHRS system is controlled using a small Raspberry Pi that handles sensor initialization and datalogging. Lastly, the HOBO U20 logs internally and the Sparv Embedded Windsond data are telemetered back to the R/V CE and logged onboard. Atmospheric sensor accuracy specifications are presented in Table 1.

### 2.2. Field Experiment

NWIPS was tested in a field experiment that took place 6–8 km offshore of Little River, SC in October 2022. The objectives of the experiment were to test and evaluate the NWIPS: (i) to test the NWIPS including its various components (e.g., temperature and humidity sensors) and (ii) assess the performance of NWIPS wave-coherent measurements. Objective (i) corresponds directly to the accuracy and longevity of the SHT-85 sensors onboard the platform, especially at the lowest measurement positions, and how quickly sensor accuracy deteriorates or ceases operating due to salt fouling [12]. Furthermore, we sought to understand platform contamination including the efficacy of the windsock-based orientation system. Measurements were performed over ~2 h. However, due to technical issues with onboard R/V CE vessel positioning data, only 80 min of NWIPS data are used in this manuscript. Position and heading data are required to correct wind speed (and direction) measured by a Vaisala WXT536 onboard the R/V CE, and wind speed is needed for the bulk-to-skin sea-surface temperature corrections. Thus, a complete vertical temperature profile is only possible when these corrections can be made. Additionally, two of the Windsonds failed due to issues with the telemetry system, so only two upper altitude measurements from the weather balloon are used in this manuscript (those at nominally 7 m and 15 m). Scalar profiles were sampled at 1 Hz providing a total of 4800 instantaneous vertical profiles. During the experiment, the buoy remained within 30−160 m of the research vessel.

Supplementary meteorological measurements were obtained from a shipboard-mounted Vaisala WXT536 weather station. During the experiment, these meteorological measurements did not vary extensively. Atmospheric stability $\left(\frac{z}{L}\right)$ is estimated to be in the unstable regime ($-0.72<\frac{z}{L}<-0.27$), where z = 2.37 m (the altitude of measurements) and L is the Obukhov length defined as [20]:

(1) $$L=\;\frac{-u_{*0}{}^{3}}{[\kappa \left(\frac{g}{\theta_{v}}\right)\bar{w^{\prime }\theta_{v}^{\prime }}}\;$$

where $u_{*0}$ is the friction velocity at the surface, κ the von Karman constant, g acceleration due to gravity, $\theta_{v}$ mean virtual potential temperature, $w^{\prime }$ the turbulent vertical wind component, and ${\theta_{v}}^{\prime }$ the turbulent virtual potential temperature. The Coupled Ocean-Atmosphere Response Experiment (COARE; [21,22]) bulk algorithm is used to estimate the Obukhov length. The general wind direction for the duration of the experiment was north to northwest. The air temperature varied between 17–19 °C and the water temperature was relatively constant at ~22 °C. Wind speed was observed to be between 1 ms^−1^ and 6 ms^−1^ and relative humidity between ~50% and ~65%.

The measured bulk sea-surface temperature ($T_{B}$) is corrected to skin temperature by the process detailed in [23]. Skin temperature (φ) is defined for low (U < 4 m/s) and high (U≥ 4 m/s) instantaneous wind speeds as:

(2) $$\varphi_{l}=T_{B}-\Delta T_{l}-\Delta T_{local}$$

(3) $$\varphi_{h}=T_{B}-\Delta T_{h}$$

where subscripts “l” and “h” denote low and high wind conditions, respectively. Correction factors $\Delta T_{l}$ and $\Delta T_{h}$ are defined as [23]:

(4) $$\Delta T_{l}=0.035U^{2}+\left(-0.24U\right)+0.85,\;U<4\;\text{m/s}$$

(5) $$\Delta T_{h}=-0.0037U+0.35,\;U\geq 4\;\text{m/s}$$

where U is wind speed, obtained here from the meteorological station (Vaisala WXT536) located onboard the R/V CE. In low-wind-speed regimes, local diurnal heating effects must also be accounted for in (2) [23]:

(6) $$\Delta T_{local}=0.11\sin\left(10.35t+0.67\right)$$

where t is the local hour in 24 h format (e.g., 1 PM = 13). Wind speed is considered the primary driver of variability in sea surface temperature for U≥4 m/s [23].

**Figure 2:**
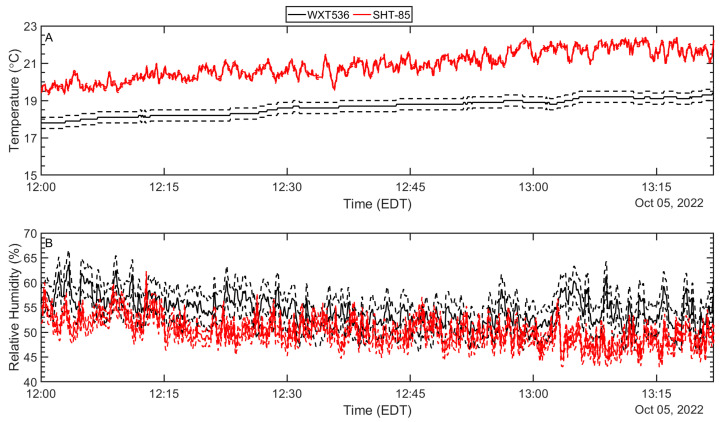
Time series of air temperature (**A**), and relative humidity (**B**) obtained aboard the R/V CE (Vaisala WXT536; black) compared to the measurements obtained from the buoy (SHT-85; red) at a similar altitude (2.37 m above the instantaneous sea-surface). Dashed lines correspond to instrument error reported by the manufacturer for each respective sensor.

The comparison of the time series of temperature and relative humidity between NWIPS and measurements performed aboard the R/V CE by the Vaisala weather station are shown in Figure 2. Measurements in Figure 2 are for similar altitudes above the instantaneous sea surface (~2.37 m) for both the weather station and NWIPS.

For the duration of the experiment, trends over time of measurements from both platforms are in relatively good agreement despite an apparent bias for temperature. Differences between air temperature measurements are within 2.5 °C with a root mean square error (RMSE) of 2.31 °C and an average difference of −2.28 °C. This bias is an order of magnitude larger than that observed under a range of wind speeds (0 m/s–10 m/s) inside a small wind tunnel in controlled laboratory conditions. However, when in close proximity during the deployment preparation on-station, a bias (average difference) of—1.5 °C was observed—at least partly explaining the bias observed in Figure 2. Because the bias was not observed during wind-tunnel testing, we conclude that the observed bias is likely related to differences in radiation—how it is mitigated by each sensor and/or differences in incoming radiation due to sensor positioning.

There are several possible sources of uncertainty stemming from discrepancies in the radiation itself or how it is mitigated. First, the type of radiation shield used by the weather station differs from that used on the SHT-85 sensors (i.e., stacked plate vs. slit-style). Second, the R/V CE must be aligned with the waves for stability purposes while drifting, which on this particular day, caused the weather station to be located in the shade of the ship’s superstructure. Perhaps the difference in the radiation between the two locations was significant enough to contribute to this temperature discrepancy. Lastly, naturally ventilated weather stations under full sunlight and U < ~4 m/s can lead to inaccuracies of >±1 °C [24]. Furthermore, stacked plate radiation shields (utilized by the Vaisala weather station) can have errors between ±3 °C to ±1 °C for winds ranging from 0.5 m/s to 5 m/s, respectively [25]. Measured wind speeds during the field experiment were <5 m/s 97% of the time; thus, ventilation issues could also be contributing to the discrepancy. Finally, it should be noted that a portion of the difference could also be associated with spatial inhomogeneity.

**Figure 3:**
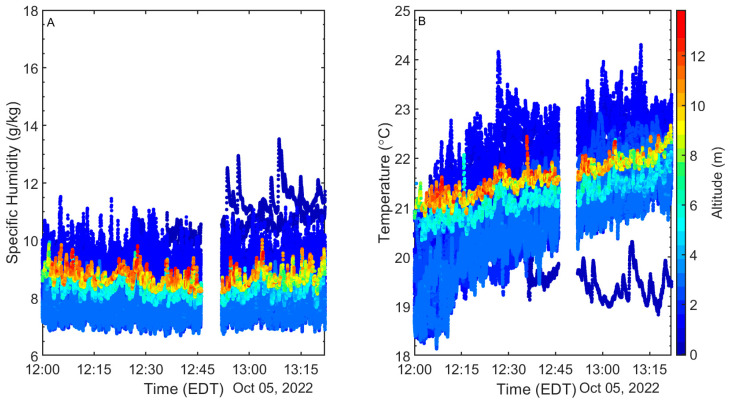
Time series of air temperature (**A**), and relative humidity (**B**) obtained from NWIPS. The color bar shows the sensors’ respective altitude.

Relative humidity, which has the greatest measurement uncertainty, has a 5.4% RMSE and an average difference of 4.26%, which is slightly higher than the cumulative error from both sources (i.e., Vaisala WXT536 relative humidity error is ±3% and the SHT85 is ±1.5%). In addition, like the temperature, these slight humidity variations between the R/V CE and NWIPS may also be at least partly a result of inhomogeneity. The time series of atmospheric sensors (mast-mounted SHT-85s and Windsonds) onboard NWIPS is presented in Figure 3.

Sea surface spectra measured during the experiment exhibited a single peak in the wind-wave frequency range. The peak wind-sea period and significant wave height (*H_s_*) were 3.7 s and 0.18 m, respectively. A qualitative assessment, via video recording, of white capping conditions indicates that whitecap coverage was <5% in the vicinity of the experiment, consistent with observed coverage in similar wind speeds [26].

Utilizing NWIPS onboard pressure sensor measurements, sea-surface displacement is obtained via hydrostatic relationship:

(7) $$\eta \left(t\right)=\frac{P_{abs}\left(t\right)-P_{atm}\left(t\right)}{\rho_{w}g}-h_{offset}$$

where $P_{abs}$ is the absolute pressure measured from the forward-located submerged pressure sensor (Figure 1), $P_{atm}$ is the atmospheric pressure measured from the weather station onboard the R/V CE, $\rho_{w}$ is water density assumed to be 1024 kg m^−3^, and $h_{offset}$ is the depth of the pressure sensor below the buoy hull mean water line.

**Figure 4:**
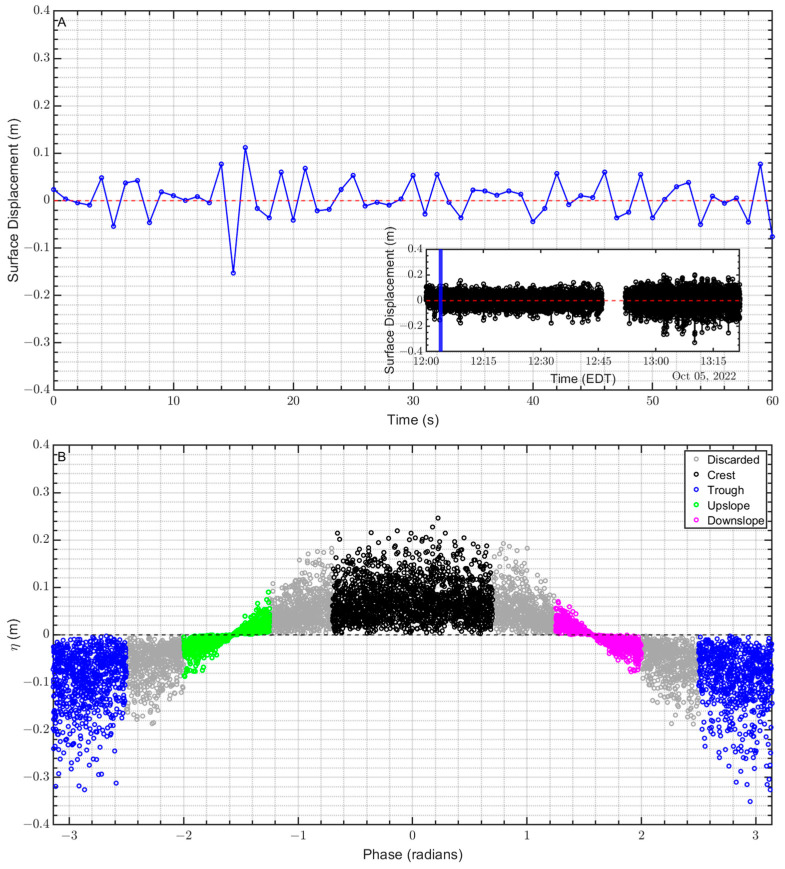
Time series of surface displacement (**A**) and sea surface displacement binned by phases estimated via Hilbert transform (**B**). The red and black dashed lines represent mean water level for (**A**,**B**), respectively. In Panel (**A**) the inset shows the entire surface displacement time series where the portion highlighted in (**A**) is marked by the blue line.

The ocean surface wave phase is computed based on a Hilbert transform of $\eta \left(t\right)$; the Hilbert transform extracts frequency components from non-linear, non-stationary time series. It is computed via a Fourier transform of the detrended sea surface displacement time series ($\eta \left(t\right))$ and suppression of Fourier coefficients associated with negative frequencies, followed by an inverse Fourier transform. This procedure results in a complex time series signal from which a phase of the original signal can be extracted [27]. The phases are then binned into four segments: crest, trough, upslope, and downslope, as shown in Figure 4.

## 3. Results

To compare vertical scalar profiles across wave phases (crest, trough, and up/down slope), a fixed coordinate system relative to the mean water level is used. The altitude of the SHT85 sensor above MWL is determined based on the SHT85 sensor height above the instantaneous water level and the measured instantaneous sea surface displacement relative to MWL. The vertical temperature and humidity profiles in each phase bin are averaged to generate phase-averaged vertical profiles relative to MWL. It is important to note that the Windsond altitudes will vary (at or below the nominal altitude) as a result of the movement of the balloon caused by the wind; thus, their exact altitude is obtained via pressure by the Windsond in conjunction with the measured surface pressure from the Vaisala WXT536. Thus, in addition to phase-averaging the Windsond data, it is also altitudinally averaged by binning the data into 0.5 m bins from 4 m up to 15 m. Each altitude bin, on average, contains 60 measurements for each wave phase.

**Figure 5:**
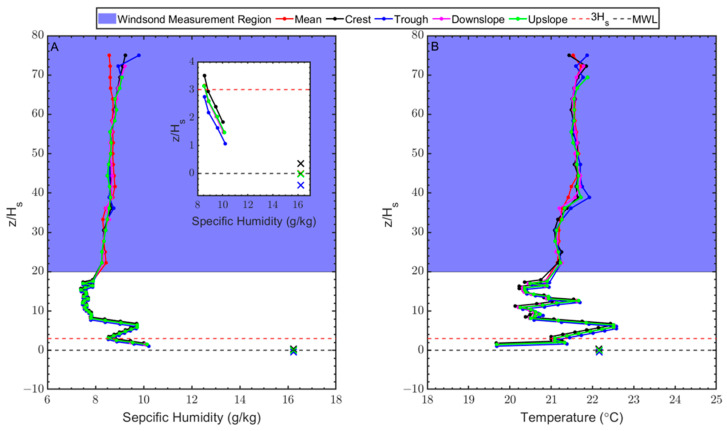
Phase-averaged vertical profiles for (**A**) specific humidity and (**B**) temperature at the crest (black), trough (blue), downslope (magenta) and upslope (green), and ~80 min average profile (red). Inset in (**A**) highlights the vertical shift of the profiles. The black dashed line represents the mean water level, whereas the red dashed line represents z ≈ $3H_{s}$. The x markers represent the sea-surface value for the corresponding profile. Altitude is normalized with significant wave height on the vertical axis.

Profiles for various wave phases are relatively similar; however, profiles are vertically shifted relative to MWL as illustrated in Figure 5. This vertical shift causes various features of the profiles to occur at different altitudes over time. The vertical shifting of the profiles is largest at the crest and trough compared to the up/down slope phases, as expected. The shifting of the profiles means that the profile in the trough of the wave extends to the surface in a similar manner as in other phases of the wave cycle. Temperature and humidity below the MWL (i.e., in the wave trough) appear to extend linearly from the values above MWL.

## 4. Summary and Discussion

This manuscript describes a measurement platform capable of high spatial and temporal sampling of temperature and humidity above a wave surface enabling measurement of wave-coherent vertical scalar profiles within the lowest altitudes of the MASL. The NWIPS consists of a 2.4 m diameter discus-type buoy with a triangular vertical mast housing 30 temperature and humidity SHT-85 sensors. These 30 sensors are vertically spaced 0.10 m apart, and when combined with a sea surface temperature probe, comprise a scalar profile that covers ~3 m in altitude from the sea surface. In addition, NWIPS utilizes a tethered balloon for up to four upper altitude measurements (with those nominally at 7 m and 15 m used in this manuscript). NWIPS is oriented into the wind via two mast-mounted windsocks to ensure measurements are taken upwind of any buoy-induced interference. A water pressure sensor is used to estimate sea surface displacement time series, and when combined with the relatively fast sampling rate of the thermodynamic sensors (1 Hz), it enables an estimation of instantaneous wave-coherent vertical distributions of temperature and humidity.

Several limitations of NWIPS have been identified from the pilot experiment. The NWIPS was developed as a proof-of-concept platform on a limited budget that is not reliable and robust enough to sustain long-term deployment. One of the needed areas of improvement is more robust electronics integration and a stable data acquisition system. These improvements would progress the reliability of NWIPS, and improved time synchronization could be accomplished through incorporation via GPS signal. Furthermore, a telemetry package would enable real-time measurement evaluation and provide insights on damage to the lowest altitude sensors enabling their replacement immediately upon failure, or equivalently switch over to a back-up sensor. In the pilot experiment, we approached this issue with back-up sensors (that were initially covered), but we had to guess when the original sensor may have been damaged; real-time data would enable sensor replacement to be more precise.

Although the onboard SHT-85 sensors were extensively tested against a Gill MaxiMet weather station, under a wide range of environmental conditions [17], and are cheap so that sensors can be replaced at low cost, an improved sensor housing is needed to make them durable enough to withstand harsher conditions. We speculate based on the pilot experiment that the maximum operating sea-state would be a function of the sensors and not the platform, which is why this improvement is so critical.

In terms of the data acquired, the nearby weather station on the R/V CE was useful, but a mast-mounted weather station would be ideal, particularly as it would provide a wind speed and direction at the buoy location, and also enable correction to the SHT-85 sensors as needed. In this study, surface displacement was attained via pressure measurements, but a more accurate and common method (i.e., displacement from buoy acceleration) to obtain surface displacement would be better. To obtain displacement from acceleration, experimental testing to obtain response amplitude operators (RAOs) of NWIPS is required to accurately evaluate the heave motion of the buoy. With known RAOs, the existing onboard AHRS system would be able to provide surface displacement accurately.

During the pilot experiment, both the feasibility of obtaining wave-coherent measurements and NWIPS sensor longevity and logistics were tested. Comparisons to temperature and humidity measurements made with a weather station aboard the R/V CE showed reasonable agreement. Wave phase-averaged scalar profiles were very similar between wave phases. The observations show scalar profiles shift with the displacement of the sea surface. The NWIPS platform is planned to be improved and deployed in the future over a wider range of conditions to examine how some of the preliminary trends presented here may vary with the sea state.

## Figures and Tables

**Table 1:** Manufacturer accuracy specifications for sensors. τ is sensor response time.

	Sensirion SHT-85	Sparv Embedded Windsond (S1H3)	Vaisala WXT536
Temperature	±0.1 °C τ = 5 s	±0.2 °C τ = 6 s	±0.3 °C τ = not provided
Relative Humidity (RH)	±1.5% RH τ = 8 s	±1.8% τ = 6 s	±3% at 0–90% RH ±5% at 90−100% RH τ = not provided
Pressure	-	±1 mb	±1 mb
Wind Speed	-	-	±3% at 10 m/s τ = 0.25 s
Wind Direction	-	-	±3.0° at 10 m/s

## Data Availability

The data presented in this manuscript are contained within the article’s Supplementary Materials.

## Supplementary Materials

The following supporting information can be downloaded at: https://www.mdpi.com/article/10.3390/s23084099/s1, Supplementary Materials see Data Availability Statement below. Metadata for the supporting information are contained within the supplementary materials.
